# Evidence for Suppression of Onchocerciasis Transmission in Bioko Island, Equatorial Guinea

**DOI:** 10.1371/journal.pntd.0004829

**Published:** 2016-07-22

**Authors:** Laura Moya, Zaida Herrador, Thuy Huong Ta-Tang, Jose Miguel Rubio, Maria Jesús Perteguer, Ana Hernandez-González, Belén García, Rufino Nguema, Justino Nguema, Policarpo Ncogo, Teresa Garate, Agustín Benito, Anacleto Sima, Pilar Aparicio

**Affiliations:** 1 Jimenez Diaz Foundation, Madrid, Spain; 2 National Center for Tropical Medicine, Institute of Health Carlos III, Madrid, Spain; 3 Network Biomedical Research on Tropical Diseases (RICET in Spanish), Madrid, Spain; 4 Malaria & Other Emerging Parasitic Diseases Laboratory, National Microbiology Center, Institute of Health Carlos III, Madrid, Spain; 5 National Program for Onchocerciasis and other Filariasis Control, Ministry of Health, Malabo, Equatorial Guinea; London School of Hygiene and Tropical Medicine, UNITED KINGDOM

## Abstract

Onchocerciasis or "river blindness" is a chronic parasitic neglected tropical disease which is endemic both in mainland and insular Equatorial Guinea. We aim to estimate the current epidemiological situation of onchocerciasis in Bioko Island after vector elimination in 2005 and more than sixteen years of Community Directed Treatment with Ivermectin (CDTI) by using molecular and serological approaches for onchocerciasis diagnosis. A community-based cross-sectional study was carried out in Bioko Island from mid-January to mid-February 2014. A total of 544 study participants were recruited. A complete dermatological examination was performed and three skin snips were performed in every participant for parasitological and molecular assessments. Blood spots were also taken for determination of Ov16 IgG4 antibodies trough an “in-house” ELISA assay. Overall, we found 15 out of 522 individuals suffering any onchocerciasis specific cutaneous lesions and 16 out of 528 (3.0%) with onchocercal nodules in the skin. Nodules were significantly associated with age, being more common in subjects older than 10 years than in younger people (3.9% vs. 0%, p = 0.029). Regarding the onchocerciasis laboratory assessment, no positive parasitological test for microfilaria detection was found in the skin snips. The calculated seroprevalence through IgG4 serology was 7.9%. No children less than 10 years old were found to be positive for this test. Only one case was positive for *Onchocerca volvulus* (*O*. *volvulus*) after skin PCR. The present study points out that the on-going mass ivermectin treatment has been effective in reducing the prevalence of onchocerciasis and corroborates the interruption of transmission in Bioko Island. To our knowledge, this is the first time that accurate information through molecular and serological techniques is generated to estimate the onchocerciasis prevalence in this zone. Sustained support from the national program and appropriate communication and health education strategies to reinforce participation in CDTI activities are essential to ensure progress towards onchocerciasis elimination in the country.

## Author Summary

Onchocerciasis or “river blindness” is a chronic parasitic disease which is mainly found in Sub-Saharan Africa. Onchocerciasis is endemic in both mainland and insular Equatorial Guinea. Huge achievements have been made on onchocerciasis control in Bioko Island in the last years, and the country is moving fast towards elimination. In the new elimination context, monitoring and evaluation activities with more sensitive diagnostic tools become especially necessary in order to confirm that transmission has been interrupted. Previous data on the epidemiological situation of onchocerciasis in Bioko Island are mainly based on microfilaria (MF) skin snip assessments. We aim to create evidence towards the fact that onchocerciasis transmission might have been achieved in Bioko Island after more than sixteen years of onchocerciasis control activities by using molecular and serological technics for onchocerciasis diagnosis.

## Introduction

Onchocerciasis or "river blindness" is a chronic parasitic neglected tropical disease caused by the filarial nematode *Onchocerca volvulus* (*O*. *volvulus*). It is transmitted to humans through exposure to repeated bites of infected blackflies of the genus *Similium* [1]. Adult worms live in subcutaneous nodules and form deeper worm bundles, where fertilized females can produce, during an average of ten years, millions of embryonic larvae (microfilariae) responsible for the morbidity associated with this disease [2]. Several simulid species have been incriminated in the transmission of *O*. *volvulus*. The complex *S*. *damnosum* and, to a lesser extent, the *S*. *naevei* complex are the most frequently found in Africa and the Arabian Peninsula [1].

Onchocerciasis affects many systems and organs, but most important morbidity is due to cutaneous and ophthalmologic manifestations, with different clinical grades [3]. The presence of one or another clinical manifestation varies depending on the most prevalent parasite strain circulating in the area: blindness tends to occur more frequently in the West African areas of savannah while onchocerciasis cutaneous disease (OCD) prevails in African forest areas [1]. Itching is usually the first clinical manifestation of onchocerciasis in the skin, and may occur alone or associated with OCD. Murdoch et al. (1993) described a grading system for OCD and defined five main categories, which can coexist together: acute papular onchodermatitis, chronic papular onchodermatitis, lichenified onchodermatitis, atrophic onchodermatitis and depigmented onchodermatitis [3]. This classification and grading system allowed to highlight the linkages between the different OCD forms with onchocerciasis epidemiology in different endemic areas [4].

Traditionally, the most common method for diagnosis of onchocerciasis was the detection of microfilariae (MF) in small, superficial skin biopsies (skin snips). However, skin snip examination is not sufficiently sensitive for detection of early infections or for diagnosis in persons with low MF densities. The low sensitivity of this parasitological method makes it unreliable in hypo endemic areas. So its value is insufficient to support an epidemiological decision. More recent and sensitive approaches include antibody-based diagnostic tests and PCR. Improved methods are needed for field diagnosis of onchocerciasis, to support efforts aimed at elimination of the disease [5,6]. Actually, the updated WHO guidelines (2016) for onchocerciasis control suggest the use of molecular tools in situations where interruption of transmission is suspected [7].

Onchocerciasis is endemic to tropical regions both in Africa and Latin America and in the Yemen. In Latin America, it is found in 13 foci located in 6 different countries. In 8 of the 13 foci in the region onchocerciasis elimination and transmission interruption has been achieved thank to Onchocerciasis Elimination Program for the Americas (OEPA) efforts [8]. More than 99% of onchocerciasis infected people live in Africa. It has been estimated that 21,115,000 members of the 118,285,000 African population at risk for infection are infected. Among these, 690,000 experienced visual impairment and 220,000 were totally blind [9,10]. Between 1974 and 2002, onchocerciasis was brought under control in West Africa through the work of the Onchocerciasis Control Program (OCP), mainly focused on by vector control. OCP was supplemented by large-scale distribution of ivermectin from 1989. The African Program for the Control of Onchocerciasis (APOC) was launched in 1995, with the aim to establish sustainable community-based systems for distribution of ivermectin (CDTI) in those countries where onchocerciasis was still a public health problem [11]. As a result of the success due to sustained onchocerciasis control activities, APOC paradigm has recently changed from control to a strategy of onchocerciasis elimination ‘where feasible’ [12]. In this new context, monitoring and evaluation activities become especially necessary in order to document if the transmission has been interrupted. The new elimination goal requires new approaches for assessment of CDTI needs in areas with lower infection prevalence, that may not be efficiently identified by methods such us Rapid Epidemiological Mapping for Onchocerciasis (REMO) [13].

Onchocerciasis is an endemic disease both in mainland and insular Equatorial Guinea [14]. Onchocerciasis control activities started in 1989, mainly based on massive distribution of ivermectin, but it was only in 1998 when CDTI activities were started as part of the proposed APOC mandate [15]. Since then, ivermectin coverages on the eligible population have ranged yearly between 50% and 75% [16]. Despite the fact that ivermectin coverage was below 85%, which is the coverage needed in a sustained fashion to interrupt transmission, progress towards this goal in Bioko Island has been made. This is partially due to the success in aerial larviciding campaign in 2005, which achieved elimination of *S*. *yahense* from Bioko Island, with no evidence of vector reappearance in the following three years. No further entomological assessments have been performed after 2008 [17]. Data from 1989 showed that overall onchocerciasis prevalence (measured through MF skin snips assessment) in Bioko Island was 75.2% (range 51.9% to 87.1%). This prevalence dropped to 38.4% after eight years of treatment with ivermectin (from 1989 to 1998), according to Mas et al [15]. The most recent available data from the Equatoguinean Ministry of Health (MoH), based on MF skin snip assessment, estimated a prevalence of 0–3% in Bioko Island (2013 data). Despite these achievements, the follow-up of CDTI activities has been irregular and there is no accurate information on the current prevalence of onchocerciasis in the whole country. Thus, we aim to estimate the current epidemiological situation of onchocerciasis in Bioko Island after more than sixteen years of CDTI activities, by using molecular and serological approach for onchocerciasis diagnosis. To our knowledge, this is the first time that the current epidemiological situation of onchocerciasis in Bioko Island is estimated by using non-traditional methods for onchocerciasis diagnosis.

## Materials and Methods

### Study area

The Island of Bioko is a part of the Republic of Equatorial Guinea, which also includes Rio Muni on the mainland and the island of Annobon. It is located in the Bay of Guinea in Central Africa, about 40 km southwest of the Cameroon coast. The surface area of Bioko Island is of approximately 2,017 km2, and it is about 72 km in length. Most of its 260,000 inhabitants live in the northern part of the island. The interior of the island is covered with dense forests on the steep slopes of volcanoes and calderas. The highest peak on the island reaches 3,011 m above sea level. The island has a humid tropical environment. Mean daily maximum and minimum temperatures range between 29–32°C and 19–22°C, respectively.

### Study design and sample size

A cross-sectional study was conducted from mid-January to mid-February 2014. Sampling was carried out by multistage cluster survey. The sample size was computed using Epi-Info version 3.4.1 free software considering the following parameters: 10% hypothesized prevalence and 2% standard error. We assumed a design effect of 2 corresponding to the complex design. The initial sample size was 450. It was increased (+20%) in prevision of missing data.

n = DEFF p(1-p)/e^2, where DEFF is the design effect, e is the desired standard error and p is the prevalence

**Fig 1:**
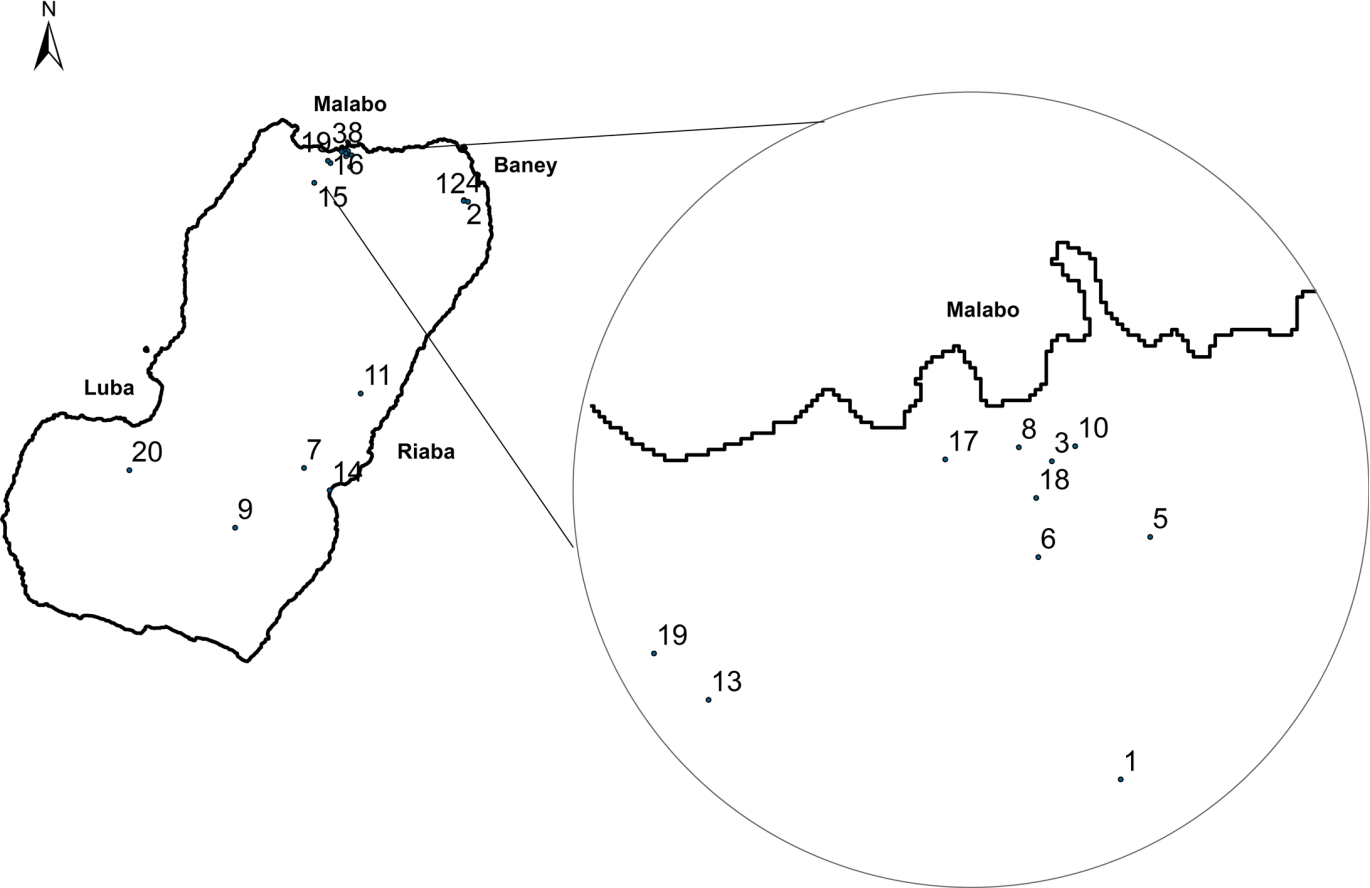
House-holds distribution in the study area, Bioko Island, Equatorial Guinea.

Firstly, twenty communities were randomly selected with probability proportional to size (Fig 1). Second sampling units were randomly selected households from an updated census from each community, provided by the head of the village (in rural areas) or neighbourhood (in urban zones). In every selected household, all individuals aged 5 years or above who had permanently lived in Bioko Island during the last five years were recruited. Children from 5 to 9 years old were included in the study in order to detect exposure to *O*. *volvulus* through Ov16, IgG4 serology test.

### Data collection

A closed ended structured questionnaire was administered to every study participant by trained medical personnel. The questionnaire was pre-tested on close areas not included in this study for clarity and cultural acceptability. It comprised the following parts: socio-demographic characteristics, risk factors for onchocerciasis and clinical data. Each interview was made by house-to-house visit. If the study participant was aged less than 15 years old, the questionnaire was answered by a parent or guardian of the teenager.

A complete dermatological examination was performed in each participant in a well-lit private room. Palpable nodules and signs of onchocercal skin disease (onchodermatitis) were assessed by trained health staff. Results were recorded on the form of “positive” or “negative”.

Three skin snips specimens were collected from every participant (two from right iliac crest, one from left iliac crest). No special collection time was considered as the microfilariae of *O*. *volvulus* are non-periodic. Two samples were immersed in normal saline solution to prevent the preparation from drying out. Then, they were sent to the local hospital laboratory to be read under a 10X microscope after 24 hours. Results were expressed for each individual as ‘positive’ or ‘negative’. Laboratory results were recorded on the original (field) registration form. The third skin snip was stored at 4°C before shipping the samples to the National Centre of Microbiology, Health Institute Carlos III (Spain), where further PCR analysis were performed.

### Laboratory techniques and microbiological determinations

#### Parasitological study

Two skin snips (one from each iliac crest) specimens were used for parasitological determination of *O*. *volvulus* MF under de 10x microscope after 24h of immersion in saline solution. The average number of MF per skin snip was obtained as well as the average number of mf from both iliac crests in each individual.

#### Serological study

Blood spots were taken from each individual for determination of Ov16 immunoglobulin G4 (IgG4) antibodies trough an “in-house” ELISA assay. The Ov16 ELISA assay uses a recombinant antigen of *O*. *volvulus* to measure prevalence of IgG4 antibodies [18,19]. Sterile techniques were used to collect blood onto the 5 x 5 cm area of Whatman 2 filter paper. The saturated blood spots were dried, individually wrapped, and transported at 4°C to the laboratory, where they were stored at −20°C until further analysis. In the laboratory, sera were eluted from filter paper punches and used in standard ELISA assay.

#### Molecular study

Preparation of the DNA template: skin snip biopsies DNA were extracted using the QIAamp DNA Mini Kit with a incubation at 56°C overnight previous to DNA extraction. DNA, was eluted with 200 μl distilled water and stored at 4°C until use.

Detection of filarial species was performed by Real Time PCR, from a modification of a Nested Filaria PCR [20]. The amplified products were purified for sequencing using Illustra DNA and Gel Band Purification Kit (General Electric Healthcare, England) and sequenced using Big Dye Terminator v3.1 Cycle Sequencing in an ABI PRISM 3700 DNA Analyzer (Applied Biosystems, U.S.A.).

### Statistical analysis

The collected data from the questionnaires and lab assessments were merged with a unique individual id and double entered into a data entry file using EpiData software, V.3.1. The data were then transferred to SPSS version 18.0 (SPSS Inc., Chicago, Illinois, USA). Frequencies, means and standard deviations (SD) were computed to summarize the data. Prevalence results with 95% confidence intervals (CI) were also calculated. Bivariate analyses by age group were performed with χ2 test for categorical data. Where a cell value was below 5, Fisher’s exact test for two–way tables was applied. The criterion for significance was set at p<0.05 based on a two-sided test.

### Ethical considerations

The study was approved by the ethical advisory boards of the Health Institute Carlos III in Spain and the Ministry of Health (MoH) in Equatorial Guinea (CEI PI 21_2014). The study complied with current national and international regulations and standards for biomedical research in human subjects. The village and neighborhood representatives were informed of the day of the visit and the scope of the study by an official letter from the Equatoguinean MoH. Written informed consent was obtained from all patients prior to study inclusion. Anonymity was assured. A written statement was also included on the introductory part of the questionnaires in which further information concerning the purpose of the study and the confidentiality of the research information was given. The written consent was obtained from parents or guardians in those individuals younger than 18 years old. Data were analysed in anonymous form.

## Results

A total of 544 study participants were recruited for the study. From them, 319 (58.6%) were females and up to two thirds (77.2%) were older than ten years. The 68.4% of the interviewed had been born in Bioko Island. The 54.0% had received none or primary education.

From the adult group (defined as aged more than 15 years old, n = 420), 43.7% were married. The 23.7% pointed out that they were working on agriculture/fishing, while 19.6% were house-keepers, 9.5% public employees and 2.7% were hand-workers.

Significant differences by sex were found for age group, marital status, occupation and the variable born in Bioko Island (Table 1).

**Table 1:** Socio-demographic characteristics of the study population, Bioko Island, Equatorial Guinea, January 2014 (n = 544).

**Variables**		**Total**		**Males**		**Females**		**p value**
		**n**	**%**	**n**	**%**	**n**	**%**	** **
Sex	Males	225	41.4	−	−	−	−	**−**
	Females	319	58.6	−	−	−	−	
Age group	5 ≤ age ≤ 10	124	22.8	69	30.7	55	17.2	p<0.001
	>10 years old	420	77.2	156	69.3	264	82.8	
Born in Bioko Island	Yes	368	68.4	163	74.1	205	64.5	0.018
	No	170	31.6	57	25.9	113	35.5	
Marital status*	Married	198	56.3	77	58.3	121	55.0	0.046
	Single	124	35.2	50	37.9	74	33.6	
	Widow	30	8.5	5	3.8	25	11.4	
Occupation *	Agriculture/fishing	87	23.7	40	29.6	47	20.3	p<0.001
	Hand worker	10	2.7	9	6.7	1	0.4	
	Public employee	35	9.5	10	7.4	25	10.8	
	House-keeper	72	19.6	0	0.0	72	31.0	
	Others	163	44.4	76	56.3	87	37.5	
Educational level	None/Primary	292	54.0	126	56.3	166	52.4	0.372
	Secondary/University	249	46.0	98	43.8	151	47.6	

* only if study participant >15 years old

### Clinical study

The 16.4% of the interviewees referred to have suffered onchocerciasis in the past. Overall, 15 out of 522 individuals pointed out suffering any onchocerciasis specific cutaneous lesions. During the clinical examination, we found that 78 out of 523 individuals (14.9%) presented itching at the time of answering the questionnaire, and 16 out of 528 (3.0%) had onchocercal nodules in the skin. The 1.3% of the study sample (7/544) showed clinical signs of onchodermatitis while 10/544 (1.8%) interviewees had any degree of leopard skin. Only 2 out of 544 presented both cutaneous manifestations (onchodermatitis and leopard skin).

After stratification by age group, we found that nodules were significantly associated with age, being more common in subjects older than 10 years than in younger people (3.9% vs. 0%, respectively, p = 0.029). Also pruritus was more frequently found in adults (17.6%) than children (5.9%, p = 0.002) (Table 2).

**Table 2:** Clinical characteristics of onchocerciasis of the study population, by age Bioko Island, Equatorial Guinea, January 2014.

Variables		**Total n**		**Children 5 ≤ age ≤ 10**		**More tan 10 years**		**p value**
		**n**	**%**	**n**	**%**	**N**	**%**	** **
**CLINICAL CHARACTERISTICS**								
Previous onchocerciasis	Yes	88	16.4	1	0.8	86	20.9	p<0.001
	No	418	77.7	117	96.7	299	72.2	
Nodules	Yes	16	3	0	0.0	16	3.9	0.029
	No	512	97	119	100.0	393	96.1	
Pruritus	Yes	78	14.9	7	5.9	71	17.6	0.002
	No	445	85.1	112	94.1	333	82.4	
Cutaneous lesions	Yes	15	2.9	1	0,8	14	3.5	0.133
	No	507	96.9	117	99,2	390	96,5	
Onchodermatitis	Yes	7	1.3	1	0.8	6	1.4	1.000
	No	537	98.7	123	99.2	414	98.6	
Leopard skin	Yes	10	1.8	0	0.0	10	2.4	0.127
	No	534	98.2	124	100.0	410	97.6	

### Parasitological study

Regarding laboratory results, none of the 544 skin snips assessments for MF detection was found positive.

### Molecular study

In parallel, 541 skin snips were submitted to specific skin PCR for *Loa loa*, *Mansonella spp* and *O*. *volvulus* (3 skin samples were missing). Skin PCR test was positive in 11/541 cases (2.0%), all of which were adults. After DNA sequencing only one case was positive for *O*. *volvulus*. The remaining ones included: seven positive cases for *Mansonella perstans*, two positive cases for *Mansonella streptocerca* and one case with *Loa Loa*, which was considered to come from blood contamination (Table 3).

**Table 3:** Laboratory assessments, Bioko Island, January 2014.

**Variables**		**TOTAL**		**Children 5 ≤ age ≤ 10**		**More than 10 years**		**P-value**
		**n**	**% (95%CI)**	**n**	**% (95%CI)**	**n**	**% (95%CI)**	
**MF in skin snips**	Negative	542	100 (CI95%: 99.3–100)	124	100 (CI95%: 97.1–100)	418	100 (CI95%: 97.1–100)	p<0.001*
	Positive	0	0 (CI95%: 0–0.7)	0	0 (CI95%: 0–2.9)	0	0 (CI95%: 0–0.9)	
**Skin PCR**	Negative	532	97.9 (CI95%: 96.4–98.9)	123	100 (CI95%: 97.1–100)	409	97.4 (CI95%: 95.4–98.6)	0.056
	Positive	11	2 (CI95%: 1.1–3.6)	0	0 (CI95%: 0–2.40)	11	2.6 (CI95%: 1.4–4.6)	
**OV16-IgG4- ELISA**	Negative	473	89.1 (CI95%: 86.1–91.5)	124	100 (CI95%: 97.1–100)	349	85.7 (CI95%: 82–89)	p<0.001
	Positive	43	8.1 (CI95%: 6.1–10.7)	0 (CI95%:0–2.38)	0 (CI95%: 0–2.9)	43	10.6 (CI95%:7.8–13.9)	
	Undefined	15	2.8 (CI95%: 1.7–4.6)	0	0 (CI95%: 0–2.9)	15	3.7 (CI95%:2.1–6)	

*Yates corrected chi square

### Serological study

Blood samples were obtained from 531 out of 544 individuals and analyzed for identification of Ov16 IgG4 antibodies by ELISA. Globally, the seroprevalence calculated was 7.9%, with and CI95%: (5.9%-10.6%). From the samples studied, 43 samples were positive and 15 undefined by OV16 -ELISA (Table 3). No children less than 10 years old were found to be positive for this test.

### Preventive practices

In relation to preventive practices among our study population, we found that 274/544 individuals (50.4%) referred that they had never taken the drug. Overall, 28% had taken ivermectin more than twice in the last five years, while 15.6% had taken it less than twice. Among those who referred to have taken ivermectin, 65.6% pointed out that they received the drug from a community distributor. When asking about the reasons for not taking ivermectin, we observed that the lack of information was the most common reason (29.9%), followed by not having suffered the disease (20.5%), age (16.5%), not being at home at the time of the drug campaign (14.3%) and lack of access to the drug (11.6%) (Table 4).

**Table 4:** Preventive treatment by age Bioko Island, Equatorial Guinea, January 2014.

Variables		**Total**		**Children 5 ≤ age ≤ 10**		**More than 10 years**		**p value**
		**n**	**%**	**n**	**%**	**n**	**%**	** **
**RISK FACTORS**								
Have you ever taken ivermectin	Yes	263	49.0	30	24.2	233	56.4	p<0.001
	No	274	51.0	94	75.8	180	43.6	
How many times in the last 5 years?	More than 2 times	151	28.0	11	8.9	140	34.0	p<0.001
	2 or less times	82	15.6	19	15.3	63	15.3	
	Never	303	50.7	94	75.8	209	50.7	
Last time of ivermectin intake*	Last year (2013/14)	111	41.7	21	70.0	90	38.1	0.002
	More than one year ago	116	43.6	9	30.0	107	45.3	
	Don´t know/don´t answer	39	14.7	0	0.0	39	16.5	
Ivermectin distributor*	Health community distributor	164	65.6	23	85.2	141	63.2	0.023
	Others	86	34.4	4	14.8	82	36.8	
Reasons for not taking ivermectin	Never had the disease	46	20.5	17	21.8	29	19.9	p<0.001
	Absent at home	32	14.3	8	10.3	24	16.4	
	Lack of information	67	29.9	12	15.4	55	37.7	
	Age	37	16.5	32	41.0	5	3.4	
	Lack of access to drug	26	11.6	7	9.0	19	13.0	
	Others	16	7.1	2	2.6	14	9.6	

* Only those who have ever taken ivermectin

Children under 10 years old referred having taken ivermectin significantly less frequently than older participants (p<0.005), while they were more likely to have taken the drug last year than those with older age (p = 0.002). Moreover, being younger was significantly associated with getting ivermectin from a community distributor (p = 0.023). The most frequent reason for not taking ivermectin in children younger than 10 years old was age (41.0%) while for adults it was mainly due to the lack of information (37.7%) (p = 0.000).

## Discussion

Our study provides evidence towards the fact that onchocerciasis transmission might have been achieved in Bioko Island after more than sixteen years of onchocerciasis control activities. For that purpose, we describe the current epidemiological, clinical and parasitological situation of onchocerciasis in Bioko Island by using molecular and serological technics to estimate onchocerciasis prevalence.

*O*. *volvulus* infection prevalence before ivermectin intervention in Bioko Island was 74.5%, and the Community Microfilarial Load (CMFL) was 28.2 microfilariae/snip [15]. In general, our results show that, even though some onchocerciasis clinical features can still be found in the population, there is a marked reduction in both the prevalence and the intensity of infection compared to the initiation of onchocerciasis control activities in the study area. No positive results after skin snip MF assessment were found, and less than 8% individuals were positive for IgG4 antibodies Ov16 by ELISA test. Furthermore, none of these cases corresponded to children under 10 years old. Only one PCR-skin test was positive for *O*. *volvulus*.

As expected, clinical and lab features were more common in adults than in younger groups. Moreover, younger group was also less likely to be involved in preventive measures. These observations also support previous evidence that repeated ivermectin treatments contribute to the reduction in transmission/prevalence of onchocerciasis as well as in the intensity of infection [21,22].

### Clinical study

Overall, we found a low number of individuals presenting clinical cutaneous manifestations of onchocerciasis in our study, and most of them were adults, as expected. Pruritus was the most common symptom, and only a few individuals pointed out suffering onchocerciasis specific cutaneous lesions (mainly leopard skin and onchodermatitis). Moreover, we found a very low number of onchocercal nodules carriers, which is coherent with the hypoendemic situation in Bioko Island.

*O*. *volvulus* transmission in Bioko Island focus has been extensively documented since 1990 to date [14]. In a survey carried out in the mid 80’ in the Island of Bioko the 28.8% of the study population presented with dermatitis, pigmentation changes and cutaneous atrophy [23]. The global prevalence of nodules was 27.2%. This research showed that onchocerciasis was widespread over the Island, with a high rate of population at risk of infection.

More recent data (1998) showed that prevalence of skin depigmentation, the proxy of longstanding infection of onchocerciasis in the community, was 9.4%, with a reduction of 7.7% after eight years of vertical ivermectin distribution [15]. In this study, it was pointed out that carriers of nodules in children aged 0–4 years decreased 2.6 times, while no significant changes were observed in other age groups [10]. This result reflected a reduction in onchocerciasis general transmission after several cycles of ivermectin distribution and vector elimination in Bioko Island.

The impact of ivermectin on OCD in onchocerciasis endemic countries has been described in the literature [24]. A multi-country study by Ozoh et al (2011) in meso and hyperendemic communities of seven study sites in Cameroon, Sudan, Nigeria and Uganda showed a substantial reduction in itching and all forms of OCD after five or six years of CDTI, including a reduction in nodules [24]. Low rates of nodule prevalence can also found in countries with long history of onchocerciasis control activities, such as most areas in Malawi, Kenya and Rwanda [25].

In our study, we observed most cases presenting itching and OCD manifestations (including nodules) were older than 10 years old, and depigmentation was exclusive of adults. Linkages between OCD and age have been well documented [24]. Age is considered to be a risk factor for depigmentation while reactive skin lesions overall have been described to be linked with younger ages [26].

### Parasitological, serological and molecular study

No positive MF skin snip assessments were found in our study population. Only one individual was found to be positive for *O*. *volvulus* in skin by PCR. Moreover, almost 8% of individuals were positive for IgG4 antibodies for Ov16 by using ELISA test. None of them were children.

Since the introduction of ivermectin distribution in 1987 in Bioko Island, an increasing number of communities have been enrolled into the treatment programme, resulting in a substantial reduction in disease prevalence [14]. Data from 1989 showed that overall prevalence (measured through MF skin snips assessment) and mean microfilarial density in Bioko Island were 75.2% (range 51.9% to 87.1%) and 32.2 mf/snip respectively [15]. Later on, Mas et al described a reduction in prevalence to 38.4% after eight years of treatment with ivermectin (from 1989 to 1998) [15]. In this study, both prevalence and intensity of infection dropped among the children under 5 years who had never been treated with an anti-filarial drug, suggesting an indirect effect of ivermectin treatment towards younger groups. Similarly, studies in Burundi [27] and Cameroon [28] have also shown a high reduction in prevalence and the intensity of microfilaridermia in young children who have never received ivermectin, but lived in four annual rounds of mass ivermectin treated communities.

The most recent available data from the Equatoguinean MoH (2013) showed a prevalence of 0–3% in Bioko Island by MF skin snip assessment. This is coherent with the parasitological results in our study.

Both microscopic detection of MF in skin snips and nodular palpation are useful epidemiological tools in the field for diagnosis and monitoring of onchocerciasis prevalence in many endemic countries [21,29–31]. Nevertheless, improved methods are needed for those areas with lower infection prevalences, which might be close to elimination [13]. Determining IgG4 antibodies for parasite-specific 16 kDa antigen (Ov16) is a recognized epidemiological tool to certificate the interruption of onchocerciasis transmission in endemic countries [32–35]. According to recent recommendations from WHO (2016), the critical threshold for interruption or elimination of transmission is the upper bound of the 95% confidence interval of less than 0.1% confirmed seropositivity to Ov-16 in children under 10 years of age [7]. In our study, the absence of positive cases for IgG4 antibodies for Ov16 by using ELISA in children younger than 10 years old suggest that the interruption of transmission might have been achieved in Bioko Island. However, the sample size should be increased to 2,000 children, in order to meet the WHO requirement needed to verify the interruption of onchocerciasis transmission [7].

Only one case was positive for *O*. *volvulus* by PCR, while the rate of onchocerciasis exposure by IgG4 antibodies was remarkably higher. The unique positive case by PCR came from a rural community in Riaba district, usually not targeted by CDTI. Surprisingly, this case´ serological test was negative, thus it might be a new infection. In a study carried out by Evans et al in Nigeria, the prevalence of onchocerciasis in children was also higher when measured by IgG4 antibodies than with skin snip. Probably, these infections were below the sensitivity of a skin snip. Other possible explanation is that their antibody response fitted a recent exposure rather than a patent one [35]. Molecular technics based on PCR have demonstrated to be more sensitive than skin snip microscopy or nodule palpation for detecting onchocerciasis [13,20]. In future research, the use of PCR in skin snips from children who are Ov16 positive may be considered as a confirmatory test.

### Preventive practices

The mentioned remarkable decrease on onchocerciasis prevalence in Bioko Island happened in spite of a moderate current involvement in preventive practices in the community. We found that more than half the population had never taken ivermectin. Among those who had taken ivermectin, most of them received the drug from a community distributor. The most important identified reason for not taking ivermectin included lack of information, followed by not suffering the disease.

Previous research from APOC (2008) highlighted a progressive increase in ivermectin therapeutic coverages in Bioko in the last years [16]. According to the most recent data from the Equatoguinean MoH, 80% of therapeutic coverage was reached during 2013 ivermectin campaign. It should be taken into account that this assessment was only performed in sentinel sites while our analysis but the whole geographical territory of Bioko Island. This might explain differences with our results.

Weaknesses in targeting younger ages (< 10 years old) were also identified as less than one fourth of the children had ever taken the drug. Within this group, age was identified as the main reason only in less than half.

Bearing in mind that ivermectin distribution campaigns target children after 5 years, our findings suggest a reduction in community participation in ivermectin campaigns as success in control program is achieved. This could be explained by a decrease on risk perception [36] or a relaxation of the control program activities due to financial constraints.

Evidence that long-term ivermectin treatment alone might interrupt and eliminate onchocerciasis in African countries has been well documented [22,34,35]. Overall, our findings support that interruption of transmission might have been achieved in Bioko Island, and interruption of ivermectin distribution could be considered in the near future. Nevertheless, decisions about ceasing CDTI activities in Bioko Island should be cautious in order to avoid recrudescence. This situation has been described in some neighbouring countries such as Cameroon after more than a decade of onchoderciasis control activities, which showed a reduction of meso- and hyperendemic onchocerciasis areas to hypoendemic areas. Nevertheless, transmission appears to continue in many areas as it was found that children under 10 years of age in the follow-up surveys had positive skin snips for MF [28]. Considerations about when to interrupt ivermectine distribution and initiate Post Treatment Survaillance (PTS) phase in Bioko Island are still unclear. Decisions on that respect will require additional entomologic studies (not performed since 2008). Moreover, extended serology studies with children of 10 years of age and under are needed in order to reach at least a 2000 children population, according to WHO guidelines (2016) [7].

### Limitations

Pre-intervention data on the prevalence and intensity of onchocerciasis infection were not available in some of the assessed communities as background information in order to assess the long-term impact of ongoing CDTI program. Moreover, the sample size was not large enough to draw definite conclusions about interruption of transmission in Bioko. Some limitations related to the comprehension level of the questionnaire among the study population should also be noted.

Regarding the Ov16 ELISA test, it is currently not known how long the IgG4 antibody response to the Ov16 antigen persists in exposed individuals and specificities vary according to authors. Cross-reactions with *Mansonella* species have been described [37]. Finally, although PCR is considered a highly sensitive test, it should be noted that cost would limit its use as regular epidemiological tool to apply on entire populations.

### Conclusions

Our findings support the idea that Equatorial Guinea is moving fast towards elimination after long term ivermectin distribution and the elimination of the vector *Simulium yahense* Bioko form from Bioko Island in 2005, among other factors. Our results will contribute to strengthen and optimize the current onchocerciasis control activities supported by the National Control Program. Appropriate communication and health education strategies to reinforce participation in CDTI activities are essential to ensure progress towards onchocerciasis elimination in the country.

## Supporting Information

S1 Checklist STROBE Checklist. (DOC)

S1 File Selected house-holds in the study area, Bioko Island, Equatorial Guinea. (DOCX)
