# Supplementary material for: Evidence for Suppression of Onchocerciasis Transmission in Bioko Island, Equatorial Guinea
Source: PLoS Negl Trop Dis. 2016 Jul 22;10(7):e0004829. doi: 10.1371/journal.pntd.0004829 (PMC4957785; doi:10.1371/journal.pntd.0004829)
Supplement: S1 File — (DOCX) [file pntd.0004829.s002.docx]

**Supporting Information**

**S1. Selected house-holds in the study area, Bioko Island, Equatorial Guinea.**

| ID | NAME |
| --- | --- |
| 1 | Santo Tomas de Aquino |
| 2 | Baney Zona Media B1 |
| 3 | Manzana Casa Bola |
| 4 | Zona Alta A1 Baney |
| 5 | Alcalde 1 Malabo |
| 6 | Colas Sesgas |
| 7 | Inasa Maule |
| 8 | GETESA |
| 9 | Moka Bioko |
| 10 | Impecsa |
| 11 | Bilelipa |
| 12 | Baney Zona Baja |
| 13 | Santa Maria 4B Malabo |
| 14 | Barrios Adyacentes Riaba |
| 15 | Sampaka 1 Malabo |
| 17 | Zona "D" C/Nº 1-25 Malabo |
| 18 | Cachirulo |
| 19 | Santa María 4A |
| 20 | Ruiché |
